# Effective German and English Language mHealth Apps for Self-management of Bronchial Asthma in Children and Adolescents: Comparison Study

**DOI:** 10.2196/24907

**Published:** 2021-05-19

**Authors:** Julian Franzmair, Susanne C Diesner-Treiber, Julian J M Voitl, Peter Voitl

**Affiliations:** 1 First Vienna Pediatric Medical Center Vienna Austria; 2 Sigmund Freud University Vienna Austria

**Keywords:** asthma, apps, mobile health, self-management, recommended apps, German, English, mobile phone

## Abstract

**Background:**

Mobile health (mHealth) apps hold great potential for asthma self-management. Data on the suitability of asthma apps intended for children are insufficient, and the availability of German language apps is still inadequate compared with English language apps.

**Objective:**

This study aims to identify functional asthma apps for children in German and to compare them with English language apps. In line with the PRISMA (Preferred Reporting Items for Systematic Reviews and Meta-Analyses) guidelines, the Google Play Store and Apple App Store are systematically searched to preselect the most efficient apps, which are then compared according to a self-compiled criteria catalog.

**Methods:**

Both app stores were screened for the term *asthma*. Following a PRISMA preselection process, the apps that met the inclusion criteria (ie, available free of charge, German or English language, and suitable for children) were rated by 3 independent persons following a criteria catalog consisting of 9 categories, some conceived for this purpose (*availability*, *child-friendly*, *learning factor*, and *range of functions*) and some adopted from existing validated catalogs (*functionality and design*, *ease of use*, *potential for improving asthma self-management*, *fun factor and incentives*, and *information management and medical accuracy*). The highest rated apps in German and English were compared.

**Results:**

A total of 403 apps were identified on the Google Play Store and the Apple App Store. Finally, 24 apps that met the inclusion criteria were analyzed. In the first step of the quality assessment, only 4 available German language asthma apps were compared with 20 English language asthma apps. The 4 German language apps were then compared with the 4 highest rated English language apps. All selected apps, independent of the language, were comparable in the following categories: *availability*, *functionality and design*, *ease of use*, and *information management and medical accuracy*. The English language apps scored significantly higher in the following categories: *potential for improving self-management*, *child-friendly*, *fun factor*, *learning factor*, and *range of function*. English language apps (mean total points 34.164, SD 1.09) performed significantly better than German language asthma apps (mean total points 22.91, SD 2.898; *P*=.003). The best rated English language app was *Kiss my asthma* (36/42 points), whereas the best rated German language app *Kata* achieved only 27.33 points.

**Conclusions:**

The recommended English language apps are *Kiss my asthma*, *AsthmaXcel*, *AsthmaAustralia*, and *Ask Me, AsthMe!,* whereas the only recommended German language app is *Kata*. The use of apps plays an increasingly important role in patients’ lives and in the medical field, making mHealth a staple in the future of asthma treatment plans. Although validated recommendations on rating mHealth apps have been published, it remains a challenging task for physicians and patients to choose a suitable app for each case, especially in non–English-speaking countries.

## Introduction

### Background

Asthma is the most common chronic disease in childhood, affecting 1 in 12 children. According to the World Health Organization, approximately 339 million [1] people worldwide are living with asthma. Asthma is not a harmless condition; according to the last World Health Organization survey in 2016, there were 417,918 deaths attributable to asthma [2]. Disease control is often difficult, especially in children, because of a poor understanding of the issue and misjudging the severity of the symptoms. According to the Global Initiative for Asthma, the treatment adjustment strategy includes education, skills training, and optimization of medications as pillars of personalized disease management. Mobile health (mHealth) apps have recently become a tool to better educate children about their illness and treatment management [3].

A systemic analysis by Farzandipour et al [4] proved the exceptional potential asthma apps have to improve the quality of life and control symptoms compared with conventional treatment methods. Routine care can benefit from functions such as medication intake reminders, symptoms tracking, transmission of peak flow measurements directly to the treating physician [5], and tailored education about the disease and its risks.

The use of apps has increased significantly in recent years and, accordingly, the use of medical apps. By 2020, the number of smartphone users worldwide is expected to reach 6.1 billion or 80% of the world’s population [6]. Approximately 62% of all smartphone owners have used their smartphone to find information about their health [7]. According to a study conducted in Hong Kong, one of the most hi-tech cities, approximately 24% of smartphone and tablet users installed a mHealth app [8]. In 2015, the available mHealth apps in the relevant stores were more than 100,000, with almost 3 trillion downloaded mHealth apps [9]. In Europe, approximately 46% of children already had a smartphone in 2019 and 41% used it every day [10]. These figures draw attention to a rising market, making further research necessary. Although the classification of mHealth apps for asthma in English has already been the focus of several studies [11-13], research has neglected German-speaking countries. Multiple tools shortlist and evaluate apps that are potentially useful in the management of chronic diseases. Some also include a guide for rating or creating a standardized user dummy, thus minimizing interindividual effects of various testers [14-16].

In Germany, more than 11 million minors are aged <14 years [17]. The prevalence of asthma among children is approximately 10% [18], indicating that the market for potential users of asthma apps for children in Germany exceeds the 1 million mark.

### Objectives

Therefore, this study aims to identify functional asthma apps in German and to compare them with English language apps. In a 3-step system, the *Google Play Store* and *Apple App Store* were systematically searched to preselect the most efficient apps using a criteria catalog, which was in part self-generated and in part adopted from existing validated catalogs. The content was further analyzed for correctness according to the current guidelines.

## Methods

### Quantitative Comparison of Apps

**Figure 1:**
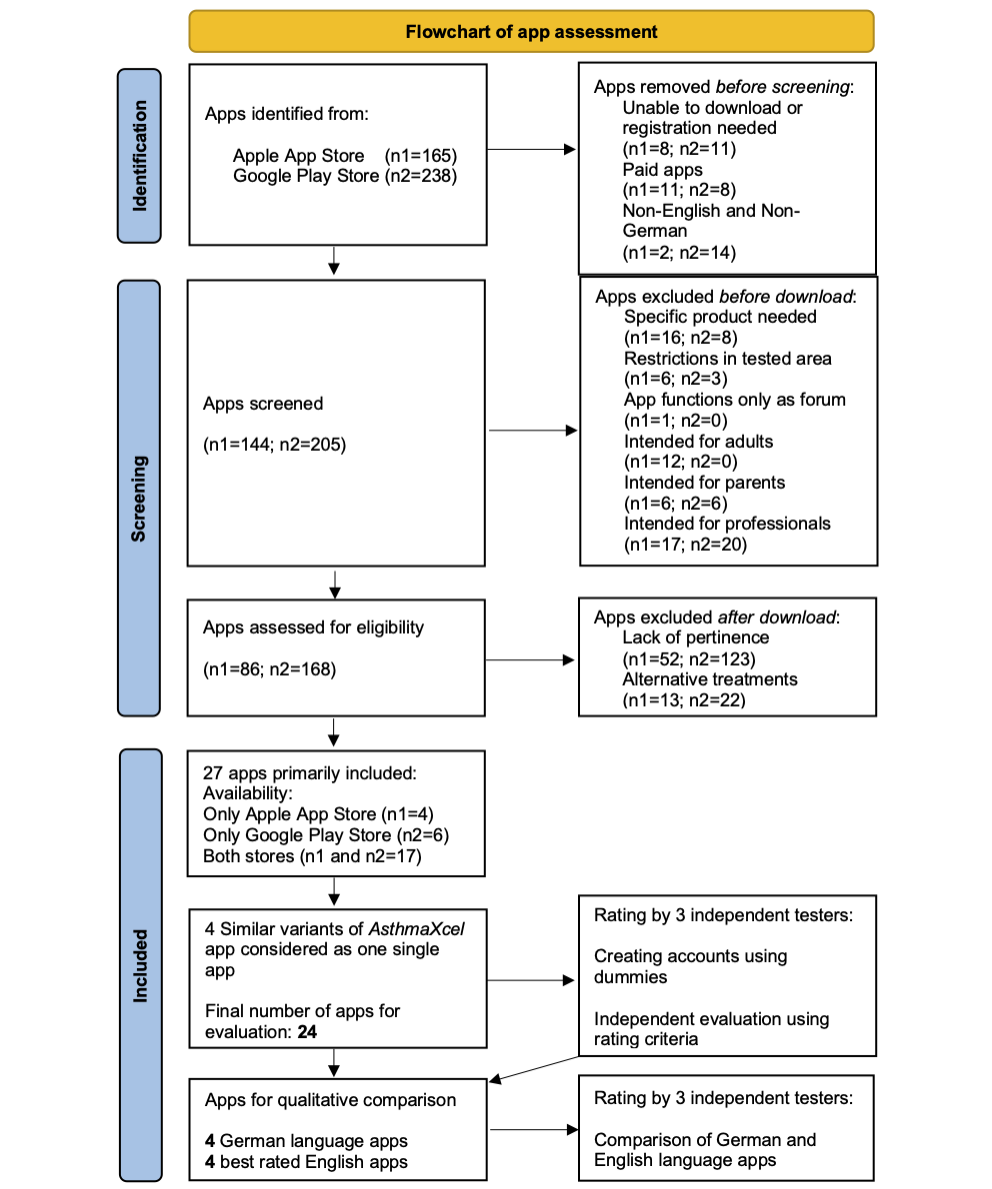
PRISMA (Preferred Reporting Items for Systematic Reviews and Meta-Analyses) flowchart of app assessment. After applying the exclusion criteria, 27 apps were primarily included. Of these, 17 were available in both stores, 4 in the Apple App Store (depicted as n1), and 6 in the Google Play Store (depicted as n2). As 1 app (AsthmaXcel) had 4 different variants, it was considered as one single app, resulting in 24 apps for the final analysis.

Two of the world’s leading mobile app platforms, the Google Play Store and Apple App Store, were used for the search. The preselection process following the PRISMA (Preferred Reporting Items for Systematic Reviews and Meta-Analyses) guidelines (Figure 1), adapted from the study by Page et al [19], was performed by one of the authors according to the inclusion and exclusion criteria. In the case of questionable results, they were checked for validity and plausibility by the other authors to ensure an objective decision.

The term *asthma* was searched in both app stores, and the apps eligible for inclusion were downloaded on a smartphone with a Google Android operating system or an Apple iPad with an iOS operating system. Inclusion (available free of charge, German or English language apps, and suitable for children and adolescents) and exclusion criteria (specific product needed; restrictions in the tested area; app forum as the only function; intended for adults, parents, or medical professionals; alternative treatments; and lack of pertinence; Multimedia Appendix 1 [3,18,20]; Figure 1) were applied to select relevant apps and compare German and English free offers for children and adolescents. As asthma affects all social classes, we only included free apps. Other studies have already pointed out that low- and middle-income populations have limited access to mHealth apps [7]. For these groups, free apps were the only viable option.

As shown in Multimedia Appendix 1, these criteria ruled out all apps irrelevant for comparison and restricted the choice to those catering to children and adolescents, those available in German and English, those available free of charge, and available following medical guidelines and recommendations.

### Qualitative Assessment of German and English Language Apps

After the preselection process, all remaining apps that met the inclusion criteria were downloaded, and their functions were evaluated by 3 independent persons following a self-compiled criteria catalog. These 3 raters tested the apps independently and without any conflicts of interest.

For an objective analysis, testing was performed following the same procedure. A private email address and contact details were used for logging in, when required, to access the functions of the apps. For a realistic approach, any minor age was selected, and, if necessary, realistic, arbitrary values about personal measurements and other data were selected.

In total, 2 test dummies (Multimedia Appendix 2) of different age groups (preschoolers and teenagers) were generated to test all available app features for each age group and gain a comprehensive insight into each app.

A total of 27 apps meeting the inclusion and exclusion criteria, suitable for use by children and adolescents, were selected for evaluation and representative comparison of their quality. The *AsthmaXcel* app with its 4 different, complementary, and overlapping variants (*AsthmaXcel*, *AsthmaXcel PRO*, *AsthmaXcel Adventures*, and *AsthmaXcelED*) was considered as one single app for the evaluation. Thus, the final number of evaluated apps was 24. The resulting app selection is presented in more detail in the *Results* section.

On authorization for this study, quality assessment was carried out by the 3 independent testers according to a criteria catalog consisting of 9 categories, some conceived for this purpose and some adopted from existing validated catalogs (Multimedia Appendix 3 [3,14-16,18,20]). The categories *availability*, *child-friendly*, *learning factor*, and *range of functions* were analyzed using self-compiled criteria. The categories *functionality and design*, *ease of use*, *potential for improving asthma self-management*, *fun factor and incentives*, and *information management and medical accuracy* were adopted from existing catalogs [14-16].

As there are no official criteria for evaluating apps, particularly for asthma apps for children, the authors designed some specifically intended to assess apps’ suitability for children and integrated them with existing standards. The child-friendliness of an app was determined based on the provider’s recommendations, the design (visual incentives for children to use the app), the range of functions (games and understandable information about asthma), engagement creation (through a reward or score system and entertaining features for children), usability (by the child alone or with parental support), and the general impression of the app. The test dummies were created to simulate a child using an app as accurately as possible (Multimedia Appendix 2).

The category *information management and medical accuracy* was analyzed using the *App Chronic Disease* *Checklist* by Anderson et al [16]. In addition, the quality of the app and its medical accuracy were checked by the authors using the Global Initiative for Asthma [3,20] and German Airway League [18] guidelines to ensure medical guidelines were met and if the recommendations (eg, instructional videos) were correct. This category was not designed to check every single guideline recommendation but rather to analyze the correctness of the provided information in general (eg, asthma medications, time point of adapting the medication, how to access medical care, and how to use an inhaler). These factors influenced the rating scores by consensus [14-16]. As 4 of the questions in this category addressed the points of information management, data protection, and app provider, this category was named *information management and medical accuracy*.

For the criteria catalog, we used a point system. Apps matching 8 categories were assigned a value between 1 and 5; those matching 1 category (availability) could score either 1 or 2 points, as illustrated in Multimedia Appendix 3. The validated criteria catalog point system differed from the one we applied to the *potential for improving asthma self-management* [14] and *fun factor and incentives* [15] categories (0-26 and 0-31 points, including half points for partial compliance) and to the *functionality and design* [16], *ease of use* [16], and *information management and medical accuracy* [16] categories (0-6 points, including half points for partial compliance). Multimedia Appendix 3 provides the ratings for each class. Classes indicate an app rating for a category with a different point system to align it with our 5-point system. The *App Chronic Disease Checklist version 1.0* according to Anderson et al [16] for the *functionality and design*, *ease of use*, and *information management and medical accuracy* [16] categories consists of 6 questions. Each response was assigned a value of 1 (full compliance), 0.5 (partial compliance), or 0 (no compliance), hence the maximum of 6 points. The points scored in the 6 questions were divided into 5 classes to ensure compatibility of the checklist protocol with our 5-point system. Half of the points were counted in the next higher class (Multimedia Appendix 3).

All 24 apps (4 versions of *AsthmaXcel* were considered as one and referred to as *AsthmaXcel*) that met the inclusion and exclusion criteria were evaluated, compared, and ranked based on the points achieved both cumulatively and in the single categories.

A total of 3 testers individually assessed the apps and then determined the mean value of points both cumulatively and in the single categories.

Therefore, apps were ranked according to the average of the total points of the 3 testers and the point system, with a maximum of 42 points. Statistical calculations on the same number of highest-ranking German and English language apps compared the quality of the apps. As only 4 of all eligible apps were in German, these were compared with the 4 highest ranked English language apps.

### Statistical Analysis

The quantitative comparison between German and English language apps considered the corresponding offers on the Google Play Store and Apple App Store. The analysis was performed using Microsoft Excel and IBM SPSS Statistics 25.0. Descriptive statistics were used to calculate and compare the results. For normally distributed results, the mean was calculated with SD, and for nonnormally distributed results, the median was determined between the maximum and minimum values. Normal distribution was tested using the Shapiro-Wilk test and confirmed for total points and all categories except *availability*, for which other variables nonparametric tests were used. The significant difference in the results was calculated using the two-tailed *t* test or, in the case of nonparametric results, using the Mann-Whitney U test. Statistical significance was set at *P<*.05.

## Results

### Quantitative Comparison of German and English Language Asthma Apps for Children and Adolescents

In total, 403 apps were identified under the term *asthma* in both app stores (Figure 1), including 238 (59.1%) on the Google Play Store and 165 (40.9%) on the Apple App Store. Of 403 apps, 27 (6.7%) met the inclusion and exclusion criteria. Of these, 17 (17/27, 63%) were available in both stores, 4 (4/27, 15%) only on the Apple App Store, and 6 (6/27, 22%) only on the Google Play Store. As the *AsthmaXcel* app has 4 different, complementary, and overlapping variants (*AsthmaXcel*, *AsthmaXcel PRO*, *AsthmaXcel Adventures*, and *AsthmaXcelED*) separately available for download, they were considered as one single app for the evaluation. Therefore, the final number of apps downloaded for the analysis was 24.

Of these 24 apps, 20 (83%) were available in English but not in German, and 4 (17%) apps were available in both German and English. None of the apps were available only in German.

The most common exclusion criterion was the lack of pertinence to the topic, although it was listed under the term asthma. The reasons for exclusion are listed in Table 1 and Figure 1.

**Table 1:** Exclusion criteria for the asthma apps.

Exclusion criteria	Google Play Store (n=215), n (%)	Apple App Store (n=144), n (%)
Lack of pertinence	123 (57.2)	52 (36.1)
Alternative treatment methods	22 (10.2)	13 (9.0)
Language	14 (6.5)	2 (1.4)
Cost	8 (3.7)	11 (7.6)
Intended for medical staff	20 (9.3)	17 (11.8)
Intended for parents	6 (2.8)	6 (4.2)
Impossible download, registration, or use	11 (5.1)	8 (5.5)
Medical device or specific product required	8 (3.7)	16 (11.1)
Asthma forum	0 (0)	1 (0.7)
Limited functions in some areas	3 (1.4)	6 (4.1)
Intended for adults	0 (0)	12 (8.3)

### Quality Assessment

All 24 apps that met the inclusion and exclusion criteria were rated by the 3 app testers. Average single category point values and total points were calculated (Table 2) and ranked based on the average total points. The calculation of the average of the points awarded by all 3 testers sometimes resulted in decimal values that were not rounded for better differentiation in the apps’ performance. Multimedia Appendix 4 shows the evaluation of each tester.

**Table 2:** Average of the 3 testers’ evaluation (individual categories and overall points of English and German language apps).

App	Categories^a^										
	Language	Availability	Functionality and design	Ease of use	Potential for improving asthma self-management	Child-friendly	Fun factor and incentives	Learning factor	Information management and medical accuracy	Range of function	Total
Maximum points	—^b^	2.0	5.0	5.0	5.0	5.0	5.0	5.0	5.0	5.0	42.0
KmAsthma	English	2.0	5.0	5.0	4.0	3.3	3.0	4.7	4.3	4.7	36.0
AsthmaXcel	English	2.0	4.0	4.3	3.3	4.7	3.3	5.0	4.0	4.0	34.6
Asthma Australia	English	2.0	4.7	3.7	3.0	5.0	2.3	4.7	3.7	4.3	33.4
Ask Me, AsthMe!	English	2.0	5.0	4.7	4.0	4.3	2.0	4.0	3.7	3.7	33.4
AsthmaMD	English	2.0	4.7	5.0	3.3	3.0	2.0	4.3	4.7	4.0	33.0
Elfy	English	2.0	4.7	4.7	2.3	3.3	1.0	4.0	4.0	3.3	29.3
Kata	German and English	2.0	5.0	3.7	3.0	2.0	1.0	2.3	5.0	3.3	27.3
Wizdypets	English	2.0	3.7	3.3	2.3	5.0	3.0	3.0	2.3	2.3	26.9
Asthmadodge	English	2.0	3.3	2.7	1.7	5.0	2.3	3.3	2.7	2.7	25.7
Asthma Eclub	English	1.0^c^	3.3	3.0	2.0	4.3	1.0	4.7	3.7	2.3	25.3
SaniQ	German and English	2.0	4.0	4.3	1.7	2.3	1.3	1.7	3.3	3.0	23.6
Asthma Tracker	German and English	2.0	3.3	3.7	1.7	2.0	1.0	1.0	3.3	2.7	20.7
Breathcount	English	1.0^d^	4.3	3.7	1.7	2.3	1.3	1.7	2.7	1.7	20.4
Allergymonitor	German and English	2.0	3.0	3.3	1.0	2.7	1.0	2.3	2.7	2.0	20.0
Rightbreath	English	1.0^c^	3.0	3.0	1.7	2.0	1.0	3.0	2.7	2.3	19.7
InhalerCounter	English	1.0^c^	3.7	3.7	1.7	1.7	1.0	1.7	2.3	2.7	19.5
AsthmaActionhero	English	1.0^c^	3.7	3.0	1.0	4.0	1.0	1.7	2.3	1.7	19.4
ASTHMA	English	1.0^c^	3.7	3.3	1.7	2.3	1.0	2.0	1.7	2.0	18.7
Asthma:Management	English	1.0^d^	1.7	3.3	1.7	1.7	1.0	3.0	2.7	1.7	17.8
mypeakflow	English	1.0^d^	3.3	2.7	1.3	1.7	1.0	1.7	3.0	1.7	17.4
Inhaler	English	1.0^d^	1.7	2.0	1.0	2.7	1.0	2.3	3.0	1.7	16.4
Peak Flow	English	1.0^d^	3.3	2.0	1.7	1.7	1.0	1.3	2.7	1.7	16.4
Asthma	English	1.0^d^	2.3	2.0	1.3	1.7	1.0	2.7	2.3	2.0	16.3
Inhaler diary	English	1.0^d^	2.7	2.3	1.0	1.3	1.0	1.3	2.0	1.3	14.0

^a^Average points of the 3 testers presented with one decimal.

^b^Cells do not add points to the scoring system but reflect the available language (English or German) and the score provided at the end after applying the total points.

^c^Apple.

^d^Android.

### Qualitative Comparison of German and English Language Asthma Apps

The best rated apps (Textbox 1) in English and German were selected and used for the statistical comparison of their quality. As only 4 of all eligible apps were in German, these were compared with the 4 highest ranked English language apps.

Quality was evaluated and compared according to the points assigned cumulatively and in single categories of the criteria catalog (Table 2).

**Four best rated apps in English and German.**

**Apps in German**

- SaniQ Asthma
- Asthma Tracker
- Kata
- AllergyMonitor

**Apps in English**

- Kiss my asthma (KmAsthma)
- AsthmaXcel
- Asthma Australia
- Ask Me, AsthMe!

### Categorial Analysis of Asthma Apps

#### Availability

As all 4 English language apps and 4 German language apps were available on the Google Play Store and the Apple App Store, they were awarded a maximum score of 2 points (median 2, minimum 2, and maximum 2) without showing any differences.

#### Functionality and Design

For the evaluation of this category, the *functionality* subitem in the *App Chronic Disease Checklist version 1.0* according to Anderson et al [16] was used. The English language apps *KmAsthma* and *Ask Me, AsthMe!* and the German language app *Kata* scored the highest (5 points). Points were deducted for other apps because of the lack of the possibility of sending data such as peak flow values and the lack of warnings for out-of-range values. The design and performance power were very satisfactory in all 8 apps; hence, all apps received at least three points in this category. The average score of all 8 apps was 4.25 (SD 0.7), the average score of apps in English was 4.7 (SD 0.41) and the average score of apps in German was 3.8 (SD 0.76). The difference between the German and English language apps was not significant (*P*=.16).

#### Ease of Use

The *App Chronic Disease Checklist version 1.0* according to Anderson et al was also used for the evaluation of this category [16]. *Km Asthma* was the only app that achieved 5 points in the *ease of use* subitem [16], as it is the only one that allows users to perform all self-management tasks easily, creates a user profile with a log-in option, and offers a reminder function. Points were deducted from all other apps for not offering these features. Nevertheless, all apps in this category performed very well, with a minimum score of 3.33, as all apps can be used offline and are intuitive. The average score for all 8 apps was 4.08 (SD 0.54), with the English language apps performing slightly, but not significantly, better (*P*=.11). The average of the English language apps was 4.4 (SD 0.49), and of the German language apps was 3.74 (SD 0.34).

#### Potential for Improving Asthma Self-management

Evaluation for this category was carried out with the help of the “Exemplary rating criteria for behavior change techniques in mHealth asthma apps” according to Abraham and Michie [14].

On the basis of the points achieved, 5 classes were conceived to align the rating in this category with the alternative 1-5-point system. None of the apps were awarded 5 points in this category. The apps *Km Asthma* and *Ask Me, AsthMe!* achieved 4 points in this category and thus the highest score among all apps. The German language app *AllergyMonitor* only achieved a minimum score of 1 point in this category. English language apps performed significantly better (*P*=.02), with an average score of 3.6 (SD 0.44), whereas German language apps’ average score was 1.8 (SD 0.72). The mean score of all 8 apps was 2.7 (SD 1.05).

#### Child-Friendly Factor

The results diverged widely in this category. Only the English language app *Asthma Australia* achieved the highest score, closely followed by *AsthmaXcel* with 4.67. A distinctive element of these 2 apps was the age-appropriate educational videos on asthma and its treatment. German language apps received less than 3 points in this category. Among these, *AllergyMonitor* was the best rated in this category, with 2.67 points. The principal reasons for points deduction were the lacking content and functions suitable for children (*Kata*, *AllergyMonitor*, *Asthma Tracker*, and *SaniQAsthma*) along with a poorly captivating design for the younger ones (*Kata* and *SaniQAsthma*). The average score was 3.28 (SD 1.15), with a significant difference (*P*=.007 between English language apps (average score 4.3, SD 0.63) and German language apps (average score 2.2, SD 0.3).

#### Fun Factor and Incentive to Use the App

This category was assessed using the “Exemplary rating criteria for gamification components in mHealth asthma apps” according to Thiebes et al [15]. On the basis of the points achieved, 5 classes were conceived to align the rating in this category with the alternative 1- to 5-point system. The app *AsthmaXcel* scored 3.33 points in this category, the highest among all apps.

The average of all 8 apps was only 1.87 (SD 0.88). Although 3 of the 4 German language apps received only 1 point, with a mean value of 1.075 (SD 0.13), the mean value of the English language apps was 2.67 (SD 0.53), indicating a significant difference between the 2 language groups (*P*=.02).

#### Learning Factor

Only *AsthmaXcel* achieved 5 points because videos about asthma and its treatment and games with questions about the topics contribute to the app’s learning factor. In this category, points were deducted because of inadequate information (*Km Asthma*, *Ask Me, AsthMe!*, and *AllergyMonitor*), no child-friendly delivery of information (*Kata*) or lacking content (*Asthma Tracker* and *SaniQAsthma*) about asthma and its treatment. The mean score of the 8 apps in this category was 3.2 (SD 1.45). The mean value of apps in German language was 1.83 (SD 0.55), whereas the mean value of apps in English language was 4.59 (SD 0.36). With *P*<.001, there was a significant difference in this category between the 2 language groups.

#### Information Management and Medical Accuracy

The accuracy of medical content is undoubtedly crucial [3,18,20]. This category was the only one with a German app (*Kata*) outperforming the English apps for following the *German Respiratory Society* website medical guidelines [18] and providing references. Points were deducted because of a lack of references (*Asthma Australia*, *Km Asthma*, and *AsthmaXcel*). An additional point was deducted for *Ask Me, AsthMe!*, *AllergyMonitor*, *Asthma Tracker*, and *SaniQAsthma* because of poor content and missing references. The average score of all 8 apps was 3.7 points (SD 0.63). The mean value of English language apps was 3.84 (SD 0.165), whereas the mean value of German language apps was 3.58 (SD 0.86). The difference in points achieved in this category by English and German language apps was not significant (*P*=.65).

#### Range of Functions

As none of the apps incorporated all the beneficial functions (general asthma information, games, diary, medication reminder, and pollen calendar), none of them achieved the highest score. The gaming app *AsthmaXcel* and the information app *Asthma Australia* lack diary and medication reminder functions, whereas diary apps (*Km Asthma*, *Ask Me, AsthMe!*, *Kata*, *AllergyMonitor*, *Asthma Tracker*, and *SaniQAsthma*) do not provide a game function.

The broadest array of functions was offered by the apps *Km Asthma* (diary and medication reminder functions, asthma action plan, and information), *AsthmaXcel* (games, information, and video quiz), and *Asthma Australia* (information, videos, quizzes, asthma control tests, and asthma action plans). These apps scored more than 4 out of 5 points. The best rated German app *Kata* (diary, medication reminder, asthma control test, inhalation instructions, pollen calendar, and general asthma information) achieved 3.33 points. Among the English language apps, most points were deducted in this category for the *Ask Me, AsthMe!* app (diary, asthma action plan, asthma control test score, inhalation videos, and inadequate general information). The German language apps *Asthma Tracker* (diary, medication reminder, and asthma control test) and *SaniQAsthma* (diary and medication reminder) scored 3 and 2.67 points, respectively, whereas *AllergyMonitor* incorporated the diary function only, thus totaling 2 points.

The mean value of the 8 apps in this category was 3.46 (SD 0.82). The mean value of the English language apps was 4.16 (SD 0.37), and the mean value of the German language apps was 2.75 (SD 0.49). The difference in points achieved between the 2 language groups was significant (*P*=.007).

#### Total Points and Overall Ranking

The evaluation of the quality of the apps using the criteria catalog determined that the English language asthma apps performed significantly better than the German language apps (Table 3).

**Table 3:** Total score of English and German language apps.

Rank	App	Language	Points, mean (SD)
1	*KmAsthma*	English	36.00 (0.82)
2	*AsthmaXcel*	English	34.00 (1.41)
3	*Asthma Australia*	English	33.33 (1.25)
4	*Ask me, Asthme!*	English	33.33 (1.25)
5	*Kata*	German	27.33 (0.94)
6	*SaniQ*	German	23.67 (1.25)
7	*Asthma tracker*	German	20.67 (4.03)
8	*Allergymonitor*	German	20.00 (2.94)

*Km Asthma* was the best rated app with 36 out of 42 points, followed by *AsthmaXcel* with 34 out of 42 points. *Km Asthma’s* unique characteristics are personalized asthma-related goals, such as medication reminders; the possibility to learn about trigger factors; and the animated, funny characters, making this app particularly suitable for children. The app also offers an asthma calendar, information about the disease, and a user-specific asthma action plan, facilitating asthma self-management. A noteworthy aspect is that *Km Asthma* and *Asthma Australia* have the same developers. *AsthmaXcel* distincts itself for its updated version incorporating both informative content and gaming components. There are 4 *AsthmaXcel* versions (*AsthmaXcel*, *AsthmaXcel PRO*, *AsthmaXcel Adventures*, and *AsthmaXcelED*). *AsthmaXcel*, *AsthmaXcel PRO*, and *AsthmaXcelED* are information apps all containing the same animated videos about asthma. *AsthmaXcel PRO* also offers virtual coins as a reward for correctly answered questions about the videos and a leaderboard ranking all users. *AsthmaXcel Adventures* is meant as a supplementary app with games designed to answer questions about other *AsthmaXcel* apps videos to get life points. This combination achieves an exceptionally high learning effect, with a focus on child-friendly education. The best rated German language app (*Kata*) achieved 27.33 out of 42 points. The app is user-friendly and has a wide range of functions (diary, medication reminder, asthma control test, inhalation instructions, pollen calendar, and general asthma information). However, the app is only partially child-friendly, and its results in the *fun factor and incentives* category were not particularly convincing.

The mean value of all the 8 apps was 28.54 points (SD 6.03). The average of the 4 English language apps was 34.165 points (SD 1.09). The average of the 4 German language apps was 22.91 points (SD 2.898). All English language apps ranked above the overall mean, whereas German language apps were below that value. The difference in the total number of points in the criteria catalog between German and English language apps was highly significant (*P*=.01).

## Discussion

### Principal Findings

This study aims to identify the quality and quantity of mHealth apps for English- and German-speaking children with bronchial asthma. Our analysis and evaluation following a criteria catalog that was in part self-compiled and in part applying analysis scores for mHealth apps [14-16] confirmed the qualitative and quantitative inequality between German and English language asthma apps. This is not surprising, as English-speaking countries are generally ahead as far as apps development is concerned. Although the variety of English language asthma apps is extensive, only a fraction of apps are available in German. The 4 apps in German language performed significantly worse than the 4 apps in English language, with an average score of 22.91 (SD 2.898) for the former versus 34.165 (SD 1.09) for the latter. The best rated English language app (*Km Asthma* 36 points) received a substantially higher score than the best rated German language app (*Kata* 27.33 points). A striking contrast was evident in 7 of the 9 categories. The German language apps only performed equally well in the *availability* category, and the *Kata* app was the best performing in the *information management and medical accuracy* category.

This study is the first to compare German and English language asthma apps for children and adolescents, and generally, little information exists about asthma apps dedicated to these age groups or, more specifically, about apps developed in German. The assortment of asthma apps on the Google Play Store and Apple App Store is large, but only a few are child-friendly, and providing comprehensive medical recommendations remains a difficult task, as none of the available options integrate all the necessary functions. As other studies also underlined, asthma self-management requires a combination of at least two apps to access all features [12,21]. Functions range from educative games to asthma diary, transmission of symptoms and peak flow values directly to the attending physician, and pollen calendars. This variety is essential, and, for children, in particular, apps that playfully educate children about asthma are recommended.

Children with high-risk asthma seem to be inclined to use asthma apps [22]. However, the search for an asthma app that pediatric patients can use on a daily basis for disease self-management in coordination with the treatment plan represents a challenge for children, adolescents, and their parents, which is why physicians rarely integrate their use. An American study refuted the concerns related to the transmission of personal health data via apps between patients and physicians [23]. Data protection of personal data is a concern for many patients and parents. Data protection regulations in English-speaking countries, such as the United States, and the European Union are quite diverse, with the European Union having more restrictive regulations. These restrictions might complicate mHealth development. As medical professionals often fail to provide exhaustive information about this tool, the choice falls on the patients or their caregivers.

However, mHealth apps are not suitable for all patient groups. Not all families might have the financial means of purchasing a smartphone for their children. Social and language barriers may hinder the use of mHealth apps. These patient groups will benefit from continuing to use conventional, manually recorded asthma diaries.

The approach to using the apps typically differs depending on age group. Primary school children will prefer playful apps that can significantly nurture their enthusiasm and motivation to learn. For instance, *AsthmaXcel* creates incentives through fun games. Currently, there are no German language apps that integrate games. The *AsthmaXcel* app was already the subject of a study about its educational aspect, but there are no confirmed results [24]. However, since the launch of its updated, more informative versions, *AsthmaXcel PRO* and *AsthmaXcel Adventures*, further studies should be conducted. *Asthma Australia* teaches children the basics of their illness using engaging and clear animations by their peers. For primary school–aged children who can already read, the *Km Asthma* and *Ask Me, AsthMe!* apps are a valid alternative as the only 2 available *diary apps*, both in German and English. Tracking asthma-related statistics with the help of a *diary app* can significantly improve disease management [25]. Regrettably, no German language app is suitable for younger primary school pupils. For older children and adolescents, the *AsthmaXcel (Adventures)* app can be recommended, as the game with questions is a useful tool for this age group. However, no gaming app for this specific age group is available in German language. As for the English language apps’ diary function, *Km Asthma* and *Ask Me, AsthMe!* are the best choice for primary school children, whereas *Km Asthma* is the best option for teenagers. *Asthma Australia* is a valid recommendation also for older children and adolescents, as the informative videos section is developed considering those specific cohorts. The *Kata* app is best suited for German speakers because it connects to the *German Respiratory Society* website through a direct in-app hyperlink, which is an excellent source of information on asthma. All 4 German language apps (*Kata*, *SaniQ*, *Asthma Tracker*, and *Allergy Monitor*) offer teenagers the option of keeping an asthma diary. These are not suitable for younger children, as they hardly offer child-friendly content. According to the developer, the *SaniQ* app is intended for users aged ≥16 years, but the App Store description recommends it from 4 years of age, therefore meeting the age-related inclusion parameter for this study.

Studies conducted on numerous diseases have already confirmed the potential of apps to support self-management and be instrumental in the treatment plan [26-28]. An increasing number of studies are focusing on the use of asthma apps [4,29-32]. Controversial research data on asthma apps’ influence on symptom improvement and exacerbation frequency reduction are also available. Symptoms appeared to improve using asthma apps [33-35], with no difference compared with placebo [33,36]. However, almost all studies investigating asthma apps included mainly adult study participants [4,10] and few adolescents [30,31,33,37-39]. A separate study concentrating on the benefits of a particular asthma app reported an improved management of the disease through the app, but with no significant difference in the control group [39]. However, the app was used by the child’s legal guardians.

Considering the growing mHealth apps market in German-speaking countries, a German version of the *Mobile App Rating Scale* [40], the *MARS-G* [41], was released in March 2020, after the final planning of our study results. Although it was not integrated into this study, *MARS-G* can be a tool for future studies on German language asthma apps.

Some studies have also evaluated the quality of asthma apps and issued recommendations for the use of suitable asthma apps [11,13,42,43]. However, data to compare the quality of asthma apps in German and English and their suitability for use by children remain lacking.

### Limitations

Although app evaluation followed objective criteria, the ratings in each category were determined by 3 persons, thus the likelihood of a biased perspective. Moreover, apps were used over a limited period, and potential updates or apps issued after the completion of this study were not included. Four apps for each language were selected as the most suitable for quality comparison; however, as the array of apps for German speakers was limited, apps in German in addition to English were included in this group.

As asthma is a disease that affects all social classes worldwide, only free-of-charge apps were included in the evaluation. This choice represents a limitation because a free app typically does not have a full range of functions and a corresponding technical implementation.

As not all apps indicate an age limit, the app provider recommendation was used to select the apps. As adolescents are considered to be children up to the age of 17 years, all apps that are not explicitly labeled as intended for adults were included. Another limiting factor is that teenagers’ preferences tend to be closer to those of adults’ preferences. Hence, the apps were rated based on their suitability for toddlers and school-aged children.

As not every app offers an asthma diary with the entry of peak flow values, the diary feature was not defined as a separate category, and warnings for out-of-range values were not tested. This aspect was included as a question in the *functionality and design* category. Separate testing of asthma diary apps should be carried out to address this limitation. As neither the Apple App Store nor the Google Play Store provides German-only apps that meet both inclusion and exclusion criteria for this study, the apps supporting other languages including German were considered eligible for comparison.

Every German language app in this study is also available in English.

### Conclusions

The use of apps plays an increasingly important role in patients’ lives and in the medical field, making mHealth a staple in the future of asthma treatment plans. Although validated recommendations on rating mHealth apps have been published, it remains a challenging task for physicians and patients to choose a suitable app for each case, especially in non–English-speaking countries. Hence, further studies are required on this topic. In addition, developers should address the necessary features’ improvements to allow a more efficient use of this tool in the future.

## Appendix

Multimedia Appendix 1 Inclusion and exclusion criteria or identification of suitable asthma apps.

Multimedia Appendix 2 Test dummies for preschoolers and teenagers.

Multimedia Appendix 3 Criteria catalog for each category and its description and rating.

Multimedia Appendix 4 Single test results of each rater.

## Abbreviations

mHealth: mobile health
PRISMA: Preferred Reporting Items for Systematic Reviews and Meta-Analyses
