# Supplementary material for: Effective German and English Language mHealth Apps for Self-management of Bronchial Asthma in Children and Adolescents: Comparison Study
Source: JMIR Mhealth Uhealth. 2021 May 19;9(5):e24907. doi: 10.2196/24907 (PMC8173395; doi:10.2196/24907)
Supplement: Multimedia Appendix 1 [file mhealth_v9i5e24907_app1.docx]

| **Multimedia Appendix 1.** Inclusion and exclusion criteria or identification of suitable asthma apps | |
| --- | --- |
| **Inclusion criteria** | **Exclusion criteria** |
| The app was available free of charge on the “Google Play Store” or the “Apple App Store” | Although listed among the results, the app was not related to asthma and categorized as deviating from the subject |
| The app supported German and English | The app recommended alternative treatment methods (such as acupressure, yoga, relaxation exercises) and therefore did not meet clinical practice guidelines (e.g., GINA guidelines, German Airway League) [3,20,18] |
| The app was suitable for children and adolescents as recommended by the provider. If the recommendation for an age group differed between the app developer and app provider, the app was included for the younger age group | The app description disclosed that it was designed for adults and not for children and adolescents. The app limits registration to adult users only |
|  | The app was intended for parents of a child with asthma |
|  | The app was intended for medical professionals and not for patients |
|  | The app was not available in Austria and Germany, or its use was limited in some regions |
|  | The app could not be downloaded or used due to technical reasons, or the registration was not possible |
|  | The app requires a medical device, or it includes only functions of a specific company device. |
|  | The app was not free of charge |
|  | The app did not support English or German |
|  | The app functions are limited to a specific region (e.g,. pollen calendar) |
|  | Asthma forum as only function of the app |
