# Supplementary material for: Effective German and English Language mHealth Apps for Self-management of Bronchial Asthma in Children and Adolescents: Comparison Study
Source: JMIR Mhealth Uhealth. 2021 May 19;9(5):e24907. doi: 10.2196/24907 (PMC8173395; doi:10.2196/24907)
Supplement: Multimedia Appendix 2 [file mhealth_v9i5e24907_app2.docx]

| **Multimedia Appendix 2.** Test dummies for preschooler and teenagers. | |
| --- | --- |
| Test dummy I - Preschooler | Test dummy II – Teenager |
| **Lorem Ipsum** (Female) | **Dolor Sit** (Male) |
| Date of Birth 11.11.2008 | Date of Birth 11.11.2015 |
| 1220 Vienna Austria | 1220 Vienna Austria |
| 155cm; 44,1kg | 113,3cm; 19,7kg |
| Literate | Supportive parents (creating account, data entry,…) |
| Adherence to doctor’s recommendations and pharmacological therapy | Adherence to doctor’s recommendations and pharmacological therapy |
| (Uncontrolled) asthma for 8 years (stage 3) | (Uncontrolled) asthma for 2 years (stage 3) |
| Enters daily readings into app (peak flow, …) | Enters daily readings into app (peak flow, …) |
| Recognizes out of range readings | Recognizes out of range readings |
| Practices sports on a regular basis | Engages in outdoor activities regularly |
| Asthma triggered by smoke, stress, pollen | Asthma triggered by dust stress, pollution, cold air, cats |
| Allergic to grass | Allergic to cats |
| No medical history besides asthma/allergy | No medical history besides asthma/allergy |
| No medication besides preventer/reliever | No medication besides preventer/reliever |
| No recent changes in medication | No recent changes in medication |
| Preventer: ICS mid dose | Preventer: ICS mid dose |
| Reliever: SABA | Reliever: SABA |
| If further clinical data was required, we used mid-range values for this age and size. If contact data was required for account confirmation, we used the study testers email address/phone number. | |
| Abbreviations: ICS: inhalative cortiocsteroide, SABA: short acting beta agonist | |
