# Supplementary material for: Effective German and English Language mHealth Apps for Self-management of Bronchial Asthma in Children and Adolescents: Comparison Study
Source: JMIR Mhealth Uhealth. 2021 May 19;9(5):e24907. doi: 10.2196/24907 (PMC8173395; doi:10.2196/24907)
Supplement: Multimedia Appendix 3 [file mhealth_v9i5e24907_app3.docx]

**Table 1.** Criteria catalog for each category and its description and rating.

| Category | Criteria description | Rating |
| --- | --- | --- |
| Availability | - Availability for download from the *Google Play Store*” or *Apple App Store* | - 1 point: availability on the *Google App Store* or the *Apple App Store* - 2 points: availability on both stores |
| Functionality and design | - Functionality and visual appeal assessment using the “App Chronic Disease Checklist version 1.0” construct functionality according to Anderson et al [16] (this construct also addresses the app design, hence the title *functionality and design* for this category) | - Class 1: 0, 0.5, or 1 point=1 point in this category - Class 2: 1.5 or 2 points=2 points in this category - Class 3: 2.5 or 3 points=3 points in this category - Class 4: 3.5 or 4 points=4 points in this category - Class 5: 4.5 or 5 points=5 points in this category |
| Ease of use | - Easy to use and suitable for children and adolescents - Assessment using the “App Chronic Disease Checklist version 1.0” construct ease of use according to Anderson et al [16] | - Class 1: 0, 0.5, or 1 point=1 point in this category - Class 2: 1.5 or 2 points=2 points in this category - Class 3: 2.5 or 3 points=3 points in this category - Class 4: 3.5 or 4 points=4 points in this category - Class 5: 4.5 or 5 points=5 points in this category |
| Potential for improving asthma self-management | - Assessment using “exemplary rating criteria for behavior change techniques in mHealth asthma apps” according to Abraham and Michie [14]. Points awarded for this category depended on the points achieved in the 26 rating criteria [14]. All 26 rating criteria were assessed, rated (maximum 26 points), divided into classes based on their ratings, and assigned 1-5 points based on the class | - Class 1: 0-6 points=1 point in this category - Class 2: 7-11 points=2 points in this category - Class 3: 12-16 points=3 points in this category - Class 4: 17-21 points=4 points in this category - Class 5: 22-26 points=5 points in this category |
| Child-friendly | - Suitable for children and adolescents - A child-friendly app should engage its users through an age-appropriate colorful design, games, videos, and animations to convey asthma information. The assessment aimed to verify the possibility for a child to use the app without help from a caregiver | - 1 point: the app was not child-friendly at all - 2 points: the app was not very child-friendly - 3 points: the app was partially child-friendly - 4 points: the app was child-friendly - 5 points: the app was very child-friendly |
| Fun factor and incentives | - Assessment using “exemplary rating criteria for gamification components in mHealth asthma apps” according to Thiebes et al [15]. Points awarded for this category depended on the points achieved in the 31 rating criteria [15]. All 31 rating criteria were assessed, rated (0: no compliance, 0.5: partial compliance, and 1 point: full compliance, maximum 31), divided into classes based on their ratings, and assigned 1-5 points based on the class | - Class 1: 0-7=1 point in this category - Class 2: 8-13 points=2 points in this category - Class 3: 14-19 points=3 points in this category - Class 4: 20-25 points=4 points in this category - Grade 5: 26-31 points=5 points in this category |
| Learning factor | - Educational impact of the app - This category refers to the range and quality of the information and its efficacy in improving knowledge about asthma, risk factors, diagnosis, and treatment. Points were awarded depending on the information quality, accuracy, and delivery | - 1 point: the app did not provide information about asthma at all - 2 points: the app provided little information about asthma - 3 points: the app provided some information about asthma - 4 points: the app informed well about asthma - 5 points: the app informed very well about asthma |
| Information management and medical accuracy | - Assessment using the “App Chronic Disease Checklist version 1.0” construct information management according to Anderson et al [16]. In addition, authors checked if the apps met the “Global Initiative for Asthma” [3,20] and “German Airway League” [18] guidelines for medical accuracy. These influenced the rating scores by consensus [14-16] | - Class 1: 0, 0.5, or 1 points=1 point in this category - Class 2: 1.5 or 2 points=2 points in this category - Class 3: 2.5 or 3 points=3 points in this category - Class 4: 3.5 or 4 points=4 points in this category - Class 5: 4.5 to 6 points=5 points in this category |
| Range of functions | - Range of functions offered within the app | - 1 point: the range of functions was very small - 2 points: the range of functions was small - 3 points: the range of functions was sufficient - 4 points: the range of functions was broad - 5 points: the range of functions was extensive |
