# Supplementary material for: Effective German and English Language mHealth Apps for Self-management of Bronchial Asthma in Children and Adolescents: Comparison Study
Source: JMIR Mhealth Uhealth. 2021 May 19;9(5):e24907. doi: 10.2196/24907 (PMC8173395; doi:10.2196/24907)
Supplement: Multimedia Appendix 4 [file mhealth_v9i5e24907_app4.docx]

| **Multimedia Appendix 4.** Single test results of each rater. | | | | | | | | | | | | |
| --- | --- | --- | --- | --- | --- | --- | --- | --- | --- | --- | --- | --- |
| **Rater 1** | | | | | | | | | | | | |
|  | Categories | | | | | | | | | |  |  |
| App | I | II | III | IV | V | VI | VII | VIII | IX | X | Total |  |
| Max. Points |  | **2** | **5** | **5** | **5** | **5** | **5** | **5** | **5** | **5** | **42** |  |
| SaniQ | Ger.,Engl. | 2 | 4 | 4 | 2 | 2 | 1 | 1 | 3 | 3 | 22 |  |
| AsthmaMD | English | 2 | 5 | 5 | 3 | 3 | 2 | 4 | 4 | 4 | 32 |  |
| Asthma tracker | Ger.,Engl. | 2 | 4 | 4 | 2 | 3 | 1 | 1 | 3 | 3 | 23 |  |
| Asthma Australia | English | 2 | 4 | 3 | 3 | 5 | 3 | 5 | 5 | 5 | 35 |  |
| Allergymonitor | Ger.,Engl. | 2 | 4 | 3 | 1 | 3 | 1 | 2 | 3 | 2 | 21 |  |
| Ask Me , AsthMe! | English | 2 | 5 | 5 | 4 | 5 | 2 | 3 | 4 | 3 | 33 |  |
| Kata | Ger.,Engl. | 2 | 5 | 4 | 3 | 2 | 1 | 2 | 5 | 4 | 28 |  |
| ASTHMAXCEL | English | 2 | 4 | 4 | 3 | 5 | 4 | 5 | 4 | 4 | 35 |  |
| Asthmadodge | English | 2 | 4 | 3 | 3 | 5 | 3 | 4 | 4 | 3 | 31 |  |
| KmAsthma | English | 2 | 5 | 5 | 5 | 4 | 3 | 5 | 4 | 4 | 37 |  |
| AsthmaActionhero | English | 1 | 3 | 3 | 1 | 3 | 1 | 2 | 2 | 2 | 18 |  |
| InhalerCounter | English | 1 | 3 | 4 | 2 | 1 | 1 | 1 | 3 | 3 | 19 |  |
| Rightbreath | English | 2 | 3 | 3 | 2 | 2 | 1 | 3 | 3 | 3 | 22 |  |
| Elfy | English | 2 | 5 | 5 | 3 | 3 | 1 | 4 | 4 | 3 | 28 |  |
| ASTHMA | English | 1 | 3 | 4 | 2 | 2 | 1 | 2 | 2 | 2 | 19 |  |
| Asthma Eclub | English | 1 | 3 | 3 | 2 | 5 | 1 | 5 | 4 | 2 | 26 |  |
| Wizdypets | English | 2 | 4 | 4 | 3 | 5 | 4 | 3 | 3 | 3 | 31 |  |
| Inhaler diary | English | 1 | 2 | 2 | 1 | 1 | 1 | 1 | 1 | 1 | 11 |  |
| Asthma | English | 1 | 2 | 2 | 1 | 2 | 1 | 2 | 3 | 2 | 16 |  |
| mypeakflow | English | 1 | 4 | 3 | 2 | 2 | 1 | 2 | 2 | 2 | 19 |  |
| Asthma:Management | English | 1 | 2 | 3 | 2 | 2 | 1 | 3 | 3 | 2 | 19 |  |
| Inhaler | English | 1 | 2 | 2 | 1 | 3 | 1 | 2 | 3 | 2 | 17 |  |
| Peak Flow | English | 1 | 4 | 3 | 2 | 2 | 1 | 1 | 3 | 2 | 19 |  |
| Breathcount | English | 2 | 5 | 5 | 2 | 3 | 2 | 2 | 3 | 3 | 27 |  |
|  |  |  |  |  |  |  |  |  |  |  |  |  |
| **Rater 2** | | | | | | | | | | | | |
| App | I | II | III | IV | V | VI | VII | VIII | IX | X | Total |  |
| Max. Points |  | **2** | **5** | **5** | **5** | **5** | **5** | **5** | **5** | **5** | **42** |  |
| SaniQ | Ger.,Engl. | 2 | 4 | 4 | 2 | 3 | 2 | 3 | 2 | 3 | 25 |  |
| AsthmaMD | English | 2 | 4 | 5 | 4 | 4 | 3 | 4 | 5 | 4 | 35 |  |
| Asthma tracker | Ger.,Engl. | 2 | 2 | 2 | 1 | 2 | 1 | 1 | 2 | 2 | 15 |  |
| Asthma Australia | English | 2 | 5 | 4 | 3 | 5 | 2 | 5 | 3 | 4 | 33 |  |
| AllergyMonitor | Ger.,Engl. | 2 | 4 | 4 | 1 | 3 | 1 | 3 | 3 | 2 | 23 |  |
| Ask Me , AsthMe! | English | 2 | 5 | 4 | 4 | 4 | 2 | 4 | 3 | 4 | 32 |  |
| Kata | Ger.,Engl. | 2 | 5 | 3 | 3 | 2 | 1 | 2 | 5 | 3 | 26 |  |
| AsthmaXcel | English | 2 | 4 | 5 | 3 | 5 | 3 | 5 | 4 | 4 | 35 |  |
| Asthmadodge | English | 2 | 4 | 3 | 1 | 5 | 3 | 2 | 2 | 3 | 25 |  |
| KmAsthma | English | 2 | 5 | 5 | 4 | 3 | 3 | 4 | 4 | 5 | 35 |  |
| AsthmaActionhero | English | 1 | 3 | 2 | 1 | 4 | 1 | 2 | 2 | 2 | 18 |  |
| InhalerCounter | English | 1 | 5 | 4 | 2 | 2 | 1 | 3 | 2 | 3 | 23 |  |
| Rightbreath | English | 2 | 3 | 3 | 2 | 2 | 1 | 3 | 3 | 2 | 21 |  |
| Elfy | English | 2 | 4 | 4 | 2 | 3 | 1 | 3 | 3 | 3 | 25 |  |
| ASTHMA | English | 1 | 3 | 3 | 2 | 2 | 1 | 2 | 2 | 2 | 18 |  |
| Asthma Eclub | English | 1 | 3 | 3 | 2 | 3 | 1 | 4 | 4 | 3 | 24 |  |
| Wizdypets | English | 2 | 4 | 4 | 3 | 5 | 4 | 4 | 3 | 3 | 32 |  |
| Inhaler diary | English | 1 | 3 | 3 | 1 | 2 | 1 | 2 | 1 | 1 | 15 |  |
| Asthma | English | 1 | 3 | 3 | 2 | 2 | 1 | 2 | 2 | 3 | 19 |  |
| mypeakflow | English | 1 | 3 | 2 | 1 | 2 | 1 | 1 | 3 | 2 | 16 |  |
| Asthma:Management | English | 1 | 2 | 3 | 2 | 1 | 1 | 3 | 3 | 2 | 17 |  |
| Inhaler | English | 1 | 2 | 2 | 1 | 2 | 1 | 2 | 3 | 2 | 16 |  |
| Peak Flow | English | 1 | 3 | 2 | 2 | 2 | 1 | 2 | 2 | 2 | 17 |  |
| Breathcount | English | 1 | 4 | 3 | 2 | 2 | 1 | 2 | 1 | 1 | 17 |  |
|  |  |  |  |  |  |  |  |  |  |  |  |  |
| **Rater 3** | | | | | | | | | | | | |
| App | I | II | III | IV | V | VI | VII | VIII | IX | X | Total |  |
| Max. Points |  | **2** | **5** | **5** | **5** | **5** | **5** | **5** | **5** | **5** | **42** |  |
| SaniQ | Ger.,Engl. | 2 | 4 | 5 | 1 | 2 | 1 | 1 | 5 | 3 | 24 |  |
| AsthmaMD | English | 2 | 5 | 5 | 3 | 2 | 1 | 5 | 5 | 4 | 32 |  |
| Asthma tracker | Ger.,Engl. | 2 | 4 | 5 | 2 | 1 | 1 | 1 | 5 | 3 | 24 |  |
| Asthma Australia | English | 2 | 5 | 4 | 3 | 5 | 2 | 4 | 3 | 4 | 32 |  |
| AllergyMonitor | Ger.,Engl. | 2 | 1 | 3 | 1 | 2 | 1 | 2 | 2 | 2 | 16 |  |
| Ask Me , AsthMe! | English | 2 | 5 | 5 | 4 | 4 | 2 | 5 | 4 | 4 | 35 |  |
| Kata | Ger.,Engl. | 2 | 5 | 4 | 3 | 2 | 1 | 3 | 5 | 3 | 28 |  |
| AsthmaXcel | English | 2 | 4 | 4 | 4 | 4 | 3 | 5 | 4 | 4 | 32 |  |
| Asthmadodge | English | 2 | 2 | 2 | 1 | 5 | 1 | 4 | 2 | 2 | 21 |  |
| KmAsthma | English | 2 | 5 | 5 | 3 | 3 | 3 | 5 | 5 | 5 | 36 |  |
| AsthmaActionhero | English | 1 | 5 | 4 | 1 | 5 | 1 | 1 | 3 | 1 | 22 |  |
| InhalerCounter | English | 1 | 3 | 3 | 1 | 2 | 1 | 1 | 2 | 2 | 16 |  |
| Rightbreath | English | 2 | 3 | 3 | 1 | 2 | 1 | 3 | 2 | 2 | 18 |  |
| Elfy | English | 2 | 5 | 5 | 2 | 4 | 1 | 5 | 5 | 4 | 31 |  |
| ASTHMA | English | 1 | 5 | 3 | 1 | 3 | 1 | 2 | 1 | 2 | 19 |  |
| Asthma Eclub | English | 1 | 4 | 3 | 2 | 5 | 1 | 5 | 3 | 2 | 25 |  |
| Wizdypets | English | 2 | 3 | 2 | 1 | 5 | 1 | 2 | 1 | 1 | 18 |  |
| Inhaler diary | English | 1 | 3 | 2 | 1 | 1 | 1 | 1 | 4 | 2 | 16 |  |
| Asthma | English | 1 | 2 | 1 | 1 | 1 | 1 | 4 | 2 | 1 | 14 |  |
| mypeakflow | English | 1 | 3 | 3 | 1 | 1 | 1 | 2 | 4 | 1 | 17 |  |
| Asthma:Management | English | 1 | 1 | 4 | 1 | 2 | 1 | 3 | 2 | 1 | 16 |  |
| Inhaler | English | 1 | 1 | 2 | 1 | 3 | 1 | 3 | 3 | 1 | 16 |  |
| Peak Flow | English | 1 | 3 | 1 | 1 | 1 | 1 | 1 | 3 | 1 | 13 |  |
| Breathcount | English | 1 | 4 | 3 | 1 | 2 | 1 | 1 | 4 | 1 | 18 |  |
| Categories I: Language; II: Availability; III: Functionality and Design; IV: Ease of Use; V: Potential for Improving Asthma Self-Management; VI: Child-friendly; VII: Fun, Factor and Incentives; VIII: Learning Factor; IX: Information Management and Medical Accuracy; X: Range of Function. Abbreviations: Engl.: English, Ger.: German; | | | | | | | | | | | | |
